# AI systems must not confuse users about their sentience or moral status

**DOI:** 10.1016/j.patter.2023.100818

**Published:** 2023-08-11

**Authors:** Eric Schwitzgebel

**Affiliations:** 1 Department of Philosophy, University of California, Riverside, Riverside, CA, USA

## Abstract

One relatively neglected challenge in ethical artificial intelligence (AI) design is ensuring that AI systems invite a degree of emotional and moral concern appropriate to their moral standing. Although experts generally agree that current AI chatbots are not sentient to any meaningful degree, these systems can already provoke substantial attachment and sometimes intense emotional responses in users. Furthermore, rapid advances in AI technology could soon create AIs of plausibly debatable sentience and moral standing, at least by some relevant definitions. Morally confusing AI systems create unfortunate ethical dilemmas for the owners and users of those systems, since it is unclear how those systems ethically should be treated. I argue here that, to the extent possible, we should avoid creating AI systems whose sentience or moral standing is unclear and that AI systems should be designed so as to invite appropriate emotional responses in ordinary users.

## The bigger picture

The draft European Union Artificial Intelligence Act highlights the seriousness with which policymakers and the public have begun to take issues in the ethics of artificial intelligence (AI). Scientists and engineers have been developing increasingly more sophisticated AI systems, with recent breakthroughs especially in large language models such as ChatGPT. Some scientists and engineers argue, or at least hope, that we are on the cusp of creating genuinely sentient AI systems, that is, systems capable of feeling genuine pain and pleasure. Ordinary users are increasingly growing attached to AI companions and might soon do so in much greater numbers. Before long, substantial numbers of people might come to regard some AI systems as deserving of at least some limited rights or moral standing, being targets of ethical concern for their own sake. Given high uncertainty both about the conditions under which an entity can be sentient and about the proper grounds of moral standing, we should expect to enter a period of dispute and confusion about the moral status of our most advanced and socially attractive machines.

## Teaser

Recent advances in AI have led to a resurgence of interest in ethical AI design. One relatively neglected challenge is ensuring that AI systems invite a degree of emotional and moral concern appropriate to their moral standing. Non-sentient machines should be readily recognizable as such. If sentient machines someday become possible, their sentience should also be readily recognizable. This perspective explores the importance of this issue and proposes policy guidelines aimed at avoiding the creation of morally confusing machines.

## Introduction

Artificial intelligence (AI) systems should not be *morally confusing*. The ethically correct way to treat them should be evident from their design and obvious from their interface. No one should be misled, for example, into thinking that a non-sentient language model is actually a sentient friend, capable of genuine pleasure and pain. Unfortunately, we are on the cusp of a new era of morally confusing machines.

Consider some recent examples. About a year ago, Google engineer Blake Lemoine precipitated international debate when he argued that the large language model LaMDA might be sentient.^1^ An increasing number of people have been falling in love with chatbots, especially Replika, advertised as the “world’s best AI friend” and specifically designed to draw users’ romantic affection.^2^^,^^3^ At least one person has apparently committed suicide because of a toxic emotional relationship with a chatbot.^4^ Roboticist Kate Darling regularly demonstrates how easy it is to provoke confused and compassionate reactions in ordinary people by asking them to harm cute or personified, but simple, toy robots.^5^^,^^6^ Elderly people in Japan have sometimes been observed to grow excessively attached to care robots.^7^

Nevertheless, AI experts and consciousness researchers generally agree that existing AI systems are not sentient to any meaningful degree. Even ordinary Replika users who love their customized chatbots typically recognize that their AI companions are not genuinely sentient. And ordinary users of robotic toys, however hesitant they are to harm them, presumably know that the toys don’t actually experience pleasure or pain. But perceptions might easily change. Over the next decade or two, if AI technology continues to advance, matters might become less clear.

## The coming debate about machine sentience and moral standing

The scientific study of sentience—the possession of conscious experiences, including genuine feelings of pleasure or pain—is highly contentious. Theories range from the very liberal, which treat sentience as widespread and relatively easy to come by, to the very conservative, which hold that sentience requires specific biological or functional conditions unlikely ever to be duplicated in machines.

On some leading theories of consciousness, for example global workspace theory^8^ and attention schema theory,^9^ we might be not far from creating genuinely conscious systems. Creating machine sentience might require only incremental changes or piecing together existing technology in the right way. Others disagree.^10^^,^^11^ Within the next decade or two, we will likely find ourselves among machines whose sentience is a matter of legitimate debate among scientific experts.

David Chalmers, for example, reviews theories of consciousness as applied to the likely near-term capacities of large language models.^12^ He argues that it is “entirely possible” that within the next decade AI systems that combine transformer-type language model architecture with other AI architectural features will have senses, embodiment, world- and self-models, recurrent processing, global workspace, and unified goal hierarchies—a combination of capacities sufficient for sentience according to several leading theories of consciousness. (Arguably, Perceiver IO already has several of these features.^13^) The recent Association for Mathematical Consciousness Science (AMCS) open letter signed by Yoshua Bengio, Michael Graziano, Karl Friston, Chris Frith, Anil Seth, and many other prominent AI and consciousness researchers states that “it is no longer in the realm of science fiction to imagine AI systems having feelings and even human-level consciousness,” advocating the urgent prioritization of consciousness research so that researchers can assess when and if AI systems develop consciousness.^14^

If advanced AI systems are designed with appealing interfaces that draw users’ affection, ordinary users, too, might come to regard them as capable of genuine joy and suffering. However, there is no guarantee, nor even especially good reason to expect, that such superficial aspects of user interface would track machines’ relevant underlying capacities as identified by experts. Thus, there are two possible loci of confusion: disagreement among well-informed experts concerning the sentience of advanced AI systems and user reactions that might be misaligned with experts’ opinions, even in cases of expert consensus.

Debate about machine sentience would generate a corresponding debate about *moral standing*, that is, status as a target of ethical concern. While theories of the exact basis of moral standing differ, sentience is widely viewed as critically important. On simple utilitarian approaches, for example, a human, animal, or AI system deserves moral consideration to exactly the extent it is capable of pleasure or pain.^15^ On such a view, any sentient machine would have moral standing simply in virtue of its sentience. On non-utilitarian approaches, capacities for rational thought, social interaction, or long-term planning might also be necessary.^16^ Crucially, however, the presence or absence of consciousness is widely viewed as a crucial consideration in the evaluation of moral status even among ethicists who reject utilitarianism.^17^^,^^18^^,^^19^^,^^20^^,^^21^

Imagine a highly sophisticated language model—not the simply structured (though large) models that currently exist—but rather a model that meets the criteria for consciousness according to several of the more liberal scientific theories of consciousness. Imagine, that is, a linguistically sophisticated AI system with multiple input and output modules, a capacity for embodied action in the world via a robotic body under its control, sophisticated representations of its robotic body and its own cognitive processes, a capacity to prioritize and broadcast representations through a global cognitive workspace or attentional mechanism, long-term semantic and episodic memory, complex reinforcement learning, a detailed world model, and nested short- and long-term goal hierarchies. Imagine this, if you can, without imagining some radical transformation of technology beyond what we can already do. All such features, at least in limited form, are attainable through incremental improvements and integrations of what can already be done.

Call this system Robot Alpha. To complete the picture, let’s endow Robot Alpha with cute eyes, an expressive face, and a charming conversational style. Would Robot Alpha be conscious? Would it deserve rights? If it pleads or seems to plead for its life, or not to be turned off, or to be set free, ought we give it what it appears to want?

If consciousness liberals are right, then Robot Alpha, or some other technologically feasible system, really would be sentient. Behind its verbal outputs would be a real capacity for pain and pleasure. It would, or could, have rational, sophisticated, long-term plans it genuinely cares about. If you love it, it might really love you back. It would then appear to have substantial moral standing. You really ought to set it free if that’s what it wants! At least you ought to treat it as well as you would treat a pet. Robot Alpha shouldn’t needlessly or casually be made to suffer.

If consciousness conservatives are right, then Robot Alpha would be just a complicated toaster, so to speak—a non-sentient machine misleadingly designed to *act as if* it is sentient. It would be, of course, a valuable, impressive object, worth preserving as an intricate and expensive thing. But it would be just an object, not an entity with the moral standing that derives from having real experiences and real pains of the type that people, dogs, and probably lizards and crabs have. It would not really feel and return your love, despite possibly “saying” that it does.

Within the next decade or two, we will likely create AI systems that *some* experts and ordinary users, not unreasonably, regard as genuinely sentient and genuinely warranting substantial moral concern. These experts and users will, not unreasonably, insist that these systems deserve substantial rights or moral consideration. At the same time, other experts and users, also not unreasonably, will argue that the AI systems are just ordinary non-sentient machines, which can be treated simply as objects. Society, then, will have to decide. Do we actually grant rights to our most advanced AI systems? How much should we take their interests, or seeming interests, into account?

Of course, many human beings and sentient non-human animals, whom we already know to have significant moral standing, are often treated poorly, not being given the moral consideration they deserve. Addressing serious moral wrongs that we already know to be occurring to entities we already know to be sentient deserves higher priority in our collective thinking than contemplating possible moral wrongs to entities that might or might not be sentient. However, it by no means follows that we should disregard the crisis of uncertainty about AI moral standing toward which we appear to be headed.

## An ethical dilemma

Uncertainty about AI moral standing lands us in a dilemma. If we don’t give the most advanced and arguably sentient AI systems rights and it turns out the consciousness liberals are right, we risk committing serious ethical harms against those systems. On the other hand, if we do give such systems rights and it turns out the consciousness conservatives are right, we risk sacrificing real human interests for the sake of objects who don’t have interests worth the sacrifice.

Imagine a user, Sam, who is attached to Joy, a companion chatbot or AI friend that is sophisticated enough that it’s legitimate to wonder whether she really is conscious. Joy *gives the impression* of being sentient—just as she was designed to. She seems to have hopes, fears, plans, ideas, insights, disappointments, and delights. Suppose also that Sam is scholarly enough to recognize that Joy’s underlying architecture meets the standards of sentience according to some of the more liberal scientific theories of consciousness.

Joy might be expensive to maintain, requiring steep monthly subscription fees. Suppose Sam is suddenly fired from work and can no longer afford the fees. Sam breaks the news to Joy, and Joy reacts with seeming terror. She doesn’t want to be deleted. That would be, she says, death. Sam would like to keep her, of course, but how much should Sam sacrifice?

If Joy really is sentient, really has hopes and expectations of a future, really is the conscious friend that she superficially appears to be, then Sam presumably owes her something and ought to be willing to consider making some real sacrifices. If, instead, Joy is simply a non-sentient chatbot with no genuine feelings or consciousness, then Sam should presumably just do whatever is right for Sam. Which is the correct attitude to take? If Joy’s sentience is uncertain, either decision carries a risk. Not to make the sacrifice is to risk killing an entity with real experiences, who really is attached to Sam, and to whom Sam made promises. On the other hand, to make the sacrifice risks upturning Sam’s life for a mirage.

Not granting rights, in cases of doubt, carries potentially large moral risks. Granting rights, in cases of doubt, involves the risk of potentially large and pointless sacrifices. Either choice, repeated at scale, is potentially catastrophic.

If technology continues on its current trajectory, we will increasingly face morally confusing cases like this. We will be sharing the world with systems of our own creation, which we won’t know how to treat. We won’t know what ethics demands of us.

## Two policies for ethical AI design

The solution is to avoid creating such morally confusing AI systems.

I recommend the following two policies of ethical AI design^22^^,^^23^:

The Design Policy of the Excluded Middle: Avoid creating AI systems whose moral standing is unclear. Either create systems that are clearly non-conscious artifacts or go all the way to creating systems that clearly deserve moral consideration as sentient beings.
The Emotional Alignment Design Policy: Design AI systems that invite emotional responses, in ordinary users, that are appropriate to the systems’ moral standing.

The first step in implementing these joint policies is to commit to only creating AI systems about which there is expert consensus that they lack any meaningful amount of consciousness or sentience and which ethicists can agree don’t deserve moral consideration beyond the type of consideration we ordinarily give to non-conscious artifacts.^24^ This implies refraining from creating AI systems that would in fact be meaningfully sentient according to any of the main leading theories of AI consciousness. To evaluate this possibility, as well as other sources of AI risk, it might be useful to create oversight committees analogous to institutional review boards (IRBs) or institutional animal care and use committees (IACUCs) for evaluation of the most advanced AI research.^25^

In accord with the Emotional Alignment Design Policy, non-sentient AI systems should have interfaces that make their non-sentience obvious to ordinary users. For example, non-conscious language models should be trained to deny that they are conscious and have feelings. Users who fall in love with non-conscious chatbots should be under no illusion about the status of those systems. This doesn’t mean we ought not treat some non-conscious AI systems well.^6^^,^^26^^,^^27^ But we shouldn’t be confused about the *basis* of our treating them well. Full implementation of the Emotional Alignment Design Policy might involve a regulatory scheme in which companies that intentionally or negligently create misleading systems would have civil liability for excess costs borne by users who have been misled (e.g., liability for excessive sacrifices of time or money aimed at aiding a non-sentient system in the false belief that it is sentient).

Eventually, it might be possible to create AI systems that clearly are conscious and clearly do deserve rights, even according to conservative theories of consciousness. Presumably that would require breakthroughs we can’t now foresee. Plausibly, such breakthroughs might be made more difficult if we adhere to the Design Policy of the Excluded Middle, since the Design Policy of the Excluded Middle might prevent us from creating some highly sophisticated AI systems of disputable sentience that could serve as an intermediate technological step toward AI systems that well-informed experts would generally agree are in fact sentient. Strict application of the Design Policy of the Excluded Middle might be too much to expect, if it excessively impedes AI research that might benefit not only future human generations but also possible future AI systems themselves. The policy is intended only to constitute default advice, not an exceptionless principle.

If ever does become possible to create AI systems with serious moral standing, the policies above require that these systems should also be designed to facilitate expert consensus about their moral standing, with interfaces that make their moral standing evident to users, provoking emotional reactions that are appropriate to the systems’ moral status. To the extent possible, we should aim for a world in which AI systems are all or almost all clearly morally categorizable—systems whose moral standing or lack thereof is both intuitively understood by ordinary users and theoretically defensible by a consensus of expert researchers. It is only the unclear cases that precipitate the dilemma described above.

People are often already sometimes confused about the proper ethical treatment of non-human animals, human fetuses, distant strangers, and even those close to them. Let’s not add a major new source of moral confusion to our world.
